# Management of Sudden Sensorineural Hearing Loss in Multiple Sclerosis: A Comprehensive Case Report of a Patient with Bilateral Loss and Literature Review

**DOI:** 10.3390/life14010083

**Published:** 2024-01-03

**Authors:** Ikhee Kim, Hantai Kim

**Affiliations:** 1 Department of Otorhinolaryngology–Head and Neck Surgery, Konyang University College of Medicine, Daejeon 35365, Republic of Korea; kwingh7.7@gmail.com; 2 Department of Medicine, the Graduate School of Konyang University, Daejeon 35365, Republic of Korea; 3 Konyang University Myunggok Medical Research Institute, Daejeon 35365, Republic of Korea

**Keywords:** sudden sensorineural hearing loss, multiple sclerosis, bilateral hearing loss

## Abstract

In multiple sclerosis (MS), the occurrence of sudden sensorineural hearing loss (SSNHL) is considered rare, with reported cases predominantly being unilateral. Bilateral cases are even rarer. Here, we report a case of bilateral SSNHL in a 20-year-old male diagnosed with MS. The patient, undergoing corticosteroid therapy for the management of MS, additionally received an intratympanic dexamethasone injection; however, it could not achieve significant improvement. Subsequently, the systemic dosage was increased for one week, resulting in substantial hearing improvement in both ears after three months. A review of MS-related SSNHL cases from 1987 to 2022 revealed 39 ears in the literature, with only five ears showing no hearing recovery. A remarkable 87.2% exhibited restored hearing, presenting a more favorable prognosis compared with idiopathic SSNHL. Although there were slight variations in administration methods and duration, all documented treatment approaches involve systemic corticosteroids. In some instances, SSNHL manifested as the initial symptom of MS. When SSNHL occurs in MS, auditory brainstem response (ABR) tests may reveal prolonged abnormalities, making ABR testing effective in cases where MS is suspected following SSNHL. In conclusion, the treatment of MS-related SSNHL appears appropriate with systemic corticosteroids, showing a significantly superior prognosis compared with idiopathic SSNHL.

## 1. Introduction

Multiple sclerosis (MS) is a degenerative autoimmune disease affecting the central nervous system. In MS, demyelinated plaques occur in the central nervous system, leading to a heterogeneous condition with varied clinical manifestations and pathological features depending on the location of demyelinated plaques [1]. Clinical symptoms include somatosensory symptoms such as visual impairment, motor weakness, and hearing loss [2]. Pathologically, the mechanisms involve inflammation, demyelination, and axonal degeneration [3,4]. However, the precise cause of MS remains unclear [5]. The widely accepted theory suggests that MS originates as an inflammatory immune-mediated disorder characterized by autoreactive lymphocytes and progresses to involve microglial activation and chronic neurodegeneration [1,3,6].

Sudden sensorineural hearing loss (SSNHL) is defined as a rapid-onset hearing impairment of at least 30 dB at three or more consecutive frequencies within 72 hours [7]. It predominantly manifests as unilateral hearing loss, with bilateral occurrences being rare (less than 3%) [8]. Symptoms may include not only hearing loss but also complaints of ear fullness or tinnitus. Various identifiable causes are known, but idiopathic cases, where the exact cause is not well understood, are the most common (approximately 70%) [9]. There are opinions suggesting viral cochleitis and those attributing it to microvascular events [10,11]. Treatment typically involves corticosteroid systemic administration, intratympanic injection, or a combination of both. However, only about one-third of patients fully recover their hearing, so the prognosis is not favorable [8,12,13,14]. In terms of prognosis, there is a difference depending on the frequency of the loss. The course is significantly more favorable for low-tone loss compared with high-tone loss; high-tone loss often entails the persistence of residual symptoms such as tinnitus [15].

While a population-based study suggests a relatively higher risk of SSNHL in MS among various autoimmune diseases [16], SSNHL is not commonly considered a prevalent symptom of MS [17,18]. Fortunately, the prognosis appears more favorable than idiopathic SSNHL, with most case reports of SSNHL in MS reporting hearing recovery [19,20,21,22,23,24,25,26,27,28]. However, these reports mainly involve unilateral SSNHL, and there is a lack of documented cases of bilateral SSNHL in MS. This case report aims to present such a rare occurrence of bilateral loss and provide a scoping review, shedding light on the prognosis and management of SSNHL in MS.

## 2. Case Report

A 20-year-old male presented to the emergency room one week after experiencing sudden visual impairment in the right eye, accompanied by ocular pain. A brain CT revealed no remarkable abnormalities in the parenchyma. Treatment with intravenous (IV) methylprednisolone for optic neuritis was initiated. An earlier MR with angiography from another hospital had shown hyperintensity in T2 FLAIR images, prompting a comprehensive MRI including the orbit, cervical neck, and whole spine to evaluate the possibility of MS. One week after admission, left optic neuritis occurred, and an MRI revealed demyelinating lesions at T4–T5, confirming the diagnosis of MS (objective clinical evidence of 2 or more lesions). High-dose corticosteroids (prednisolone 60 mg/day) were administered and tapered after five days, resulting in visual recovery. The patient complained of intermittent numbness and back pain, but no new lesions appeared, and he maintained prednisolone at 10 mg/day.

After being diagnosed with MS, neurologists, ophthalmologists, and orthopedists began multidisciplinary treatment with regular follow-ups. Then, at 8 months after the MS diagnosis, the patient experienced sudden right-sided hearing loss, leading to consultation with the otolaryngology department. Audiological tests, including pure-tone audiometry (PTA), speech reception threshold (SRT), and speech discrimination score (SDS), were conducted. PTA measured the air conduction threshold at 250, 500, 1000, 2000, 4000, and 8000 Hz. Bone conduction thresholds were measured at 250, 500, 1000, 2000, and 4000 Hz. Although bone conduction thresholds were measured, the air-bone gap was absent, so bone conduction thresholds were not indicated in the figures. SRT, using disyllable words, determined the lowest intensity at which the patient accurately repeated 50% of the test words. SDS, using monosyllable words, involved the examiner speaking 25 words at the most comfortable loudness (MCL) level, with the patient repeating them. Correct responses earned 4%, resulting in a total score of 100%.

**Figure 1:**
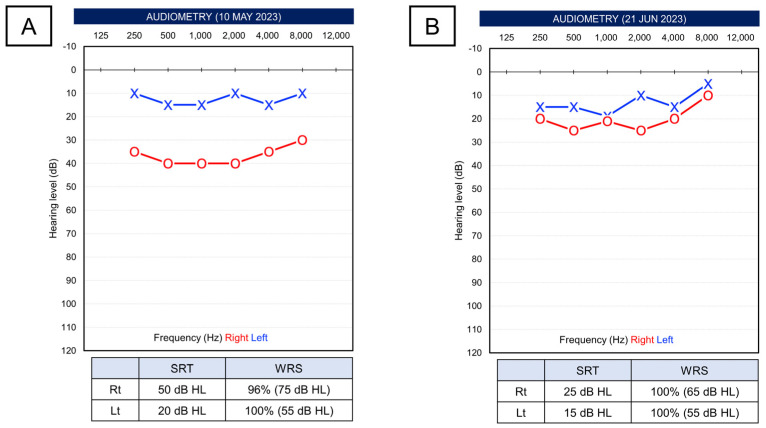
(**A**) Pure-tone thresholds and speech test results when sudden hearing loss occurred first in right ear. (**B**) After 6 weeks, the right hearing was recovered.

At the time, the average pure-tone threshold for the right ear was significantly reduced to 38.8 dB, comparable to 13.8 dB for the left ear, indicative of a clear decrease, and it corresponded to sensorineural hearing loss without an air-bone gap. SRT was reduced in the right ear to 50 dB and in the left ear to 20 dB. On the other hand, SDS was relatively better, with 96% at 75 dB stimulus in the right ear and 100% at 55 dB stimulus in the left ear (Figure 1A). The patient was maintaining a dosage of 10 mg of prednisolone at that time. Since he was taking oral prednisolone, for the additional treatment for SSNHL, a plan was made for three sessions of intratympanic dexamethasone injection (ITDI). Then, if there was no recovery after ITDIs, increasing the dose of oral corticosteroids was discussed with the neurology department. Approximately one month after the onset, the right-sided hearing completely recovered (Figure 1B). However, the patient experienced a recurrence of visual impairment, for which high-dose corticosteroid therapy was administered at another hospital. Therefore, it is possible that the symptom improvement was facilitated by the use of systemic steroids rather than by ITDI. 

**Figure 2:**
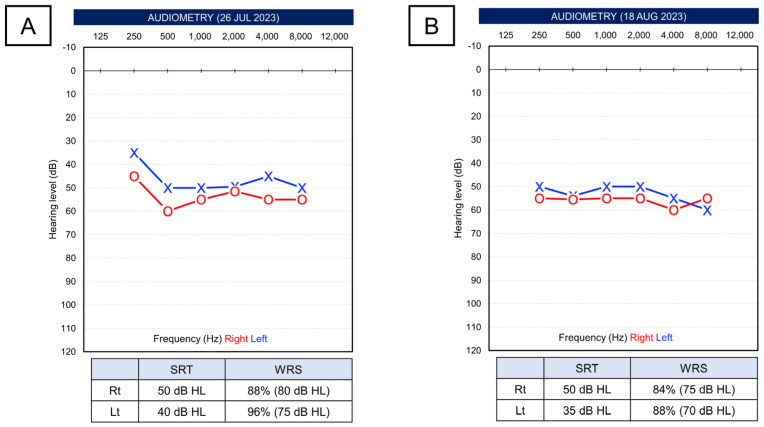
(**A**) The patient visited again with bilateral hearing loss. (**B**) Even after 3 weeks of intratympanic dexamethasone injection, pure-tone thresholds were not recovered.

**Figure 3:**
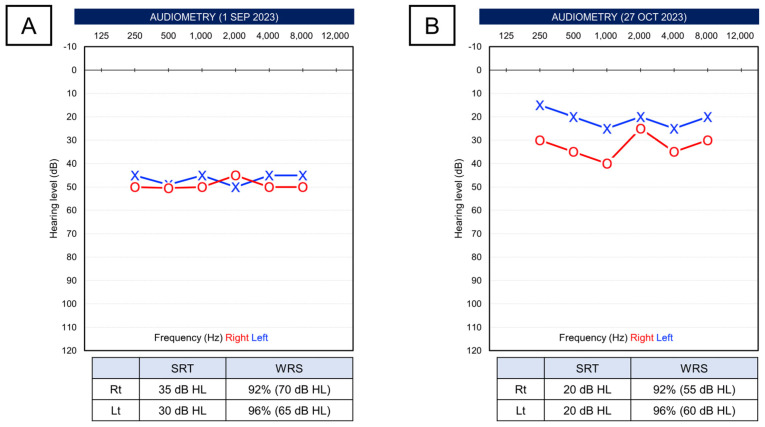
(**A**) After using the increased dose of oral methylprednisolone, speech test results showed improvement, but pure-tone thresholds were the same. (**B**) After 3 months after the occurrence of the bilateral loss, pure-tone thresholds were also significantly improved.

After approximately one month of recovering hearing in the right ear, the patient presented with sudden bilateral hearing loss. In PTA, the average thresholds were reduced, with the right ear at 55.0 dB and the left ear at 48.8 dB. SRT was 50 dB in the right ear and 40 dB in the left ear. SDS was 88% at 80 dB stimulus in the right ear and 96% at 75 dB stimulus in the left ear (Figure 2A). The patient was maintaining a dosage of 10 mg of prednisolone, and it was decided to perform three sessions of ITDI for each ear, keeping the same regimen as the previous treatment without increasing the oral corticosteroid dosage. However, there were no significant changes immediately after the treatment (Figure 2B). Subsequently, the prednisolone dosage was increased to 20 mg for an additional week. Despite minimal improvement in pure-tone thresholds (right ear 48.8 dB, left ear 47.5 dB), there was some improvement in SRT (right ear 35 dB, left ear 30 dB) (Figure 3A). After this, the patient was observed without additional treatment. Fortunately, three months after the onset, substantial recovery in hearing was noted, with pure-tone thresholds at 33.8 dB in the right ear and 22.5 dB in the left ear, and SRT at 20 dB bilaterally. SDS was 92% in the right ear at 55 dB stimulus and 96% in the left ear at 60 dB stimulus.

## 3. Discussion

The literature on SSNHL in MS primarily consists of case reports or case series involving four or fewer individuals. Notably, only two publications focused on a relatively larger sample size. Hellmann et al. analyzed 11 cases of SSNHL in MS, reporting that seven individuals experienced hearing loss as an initial symptom of MS, while the remaining four developed sudden hearing loss after an MS diagnosis (Table 1) [23]. Among the 11 cases, three exhibited spontaneous recovery within one week without specific treatment, while the other eight received high-dose IV methylprednisolone for 3–5 days, followed by a tapering course of oral prednisolone over two weeks. Only one case showed no hearing recovery, while the remaining 10 (90.1%) demonstrated substantial hearing improvement within one to two months, indicating a highly favorable outcome. Leite et al. presented seven cases of hearing loss in MS, where two individuals experienced hearing loss as the initial symptom of MS, while the others developed it within three to nineteen years after MS diagnosis (Table 1) [29]. Notably, three out of five cases with post-diagnosis hearing loss received no specific treatment, with one showing no recovery and the other two achieving partial recovery. Among the four cases treated with corticosteroids, three experienced complete recovery, and one showed no improvement. Considering the favorable outcomes observed in MS-related SSNHL compared with idiopathic SSNHL, where approximately one-third of cases exhibit no treatment response, the distinct treatment response in MS-related SSNHL appears noteworthy [8,12,13,14].

The treatment methods and outcomes of cases involving four or fewer patients were summarized in Table 2 and Table 3. Table 2 encompasses cases where sudden hearing loss occurred as the initial symptom of MS, totaling 10 ears (including one sequential bilateral case) among nine patients [19,21,26,30,31,32,33]. Treatment predominantly involved corticosteroid administration, with variations in dosage and duration. The overall outcomes were deemed favorable. All ears, except two, showed recovery within a range of 4 days to 8 months. Table 3 compiled cases where sudden hearing loss occurred in individuals already diagnosed with MS, comprising eight ears across seven patients (one case involved a simultaneous bilateral occurrence) [20,22,25,27,31,33]. Hearing fully recovered in seven ears, while one ear exhibited partial recovery. The recovery period ranged from 4 weeks to 5 months. Treatment strategies aligned with those in the preceding Table 2 cases, emphasizing corticosteroid therapy as the primary approach, albeit with slight variation in duration and dosage.

Based on our case, the two previously discussed articles (Table 1), and the literature compiled in Table 2 and Table 3, a total of 34 patients with SSNHL attributed to MS have been reported. While predominantly presenting as unilateral loss, five patients, including our case, had bilateral occurrences either simultaneously or sequentially, accounting for a total of 39 affected ear. Among these, only five ears did not recover, indicating that ultimately, 34 ears (87.2%) experienced restored hearing. It seems to have a more favorable outcome than idiopathic SSNHL. Whether SSNHL manifests as the initial symptom of MS or occurs in individuals already diagnosed with MS, it is believed that there is no significant difference in the overall course of progress. However, when SSNHL presents as the initial symptom, considering the possibility of MS becomes a plausible aspect of the diagnostic process. Nevertheless, regarding SSNHL as a common symptom of MS poses challenges.

There was one report in which the treatment method was not explicitly specified [27], whereas in all other cases, treatment was consistently based on corticosteroids. High doses of around 1 g per day were used [20,24,30,31], and some cases involved a gradual tapering over almost six months with prolonged usage [24]. In fact, these variations in dosage appear to stem from applying protocols used for SSNHL in their practices. Considering that, despite dosage differences, the outcomes were mostly favorable, it is reasonable to assume that treating SSNHL in MS patients using established protocols for idiopathic SSNHL would be effective. Some cases also explored additional treatments such as ITDI or hyperbaric oxygen therapy [22,24]. However, it remains uncertain whether these treatments yielded superior results compared with systemic corticosteroids. In a study by Anagnostouli et al., ITDI was initially attempted, and when hearing improvement did not occur, systemic corticosteroids were administered [24]. Similarly, in our presented case, immediate improvement was not achieved post-ITDI, but recovery became possible when the dosage of systemic corticosteroids was increased. This suggests that for SSNHL in MS, systemic use of steroids should be considered the standard treatment.

In terms of the recovery period, there were variations, with some cases reporting recovery in as little as around 5 days, while others took approximately 8 months for restoration. Notably, hearing recovery to the normal range was observed even in cases with profound hearing loss exceeding 90 dB [19,22,23,26]. However, understanding specific patterns of change in SDSs remains unclear. In the case we reported, speech discrimination showed a relatively smaller loss and tended to recover more rapidly compared with pure-tone thresholds. While some literature has reported the complete recovery of SDSs [20,27], this information is limited. It is difficult to conclude whether the relatively small size of speech score loss and quicker recovery, as observed in our case, represents a characteristic of SSNHL in MS or is a unique aspect confined to our case.

We would like to address the literature concerning bilateral SSNHL in MS. There were three studies reporting cases of sudden hearing loss occurring bilaterally, encompassing four patients [29,30,33]. Including the case we presented, the total number of patients is five, making it considerably rarer compared with the unilateral cases (36 patients). One case involved a sequential occurrence rather than a simultaneous bilateral onset [30]. A total of 10 ears were affected, with no recovery observed in three ears (recovery rate: 70%). While this appears somewhat different from the recovery rate of 94.1% observed in the unilateral cases (32 out of 34 ears), it is worth noting that one patient with bilateral involvement did not receive corticosteroid treatment [29]. Ultimately, considering the recovery observed in seven out of eight treated ears (87.5%), it seems that there is no significant difference in recovery rates between unilateral and bilateral cases.

Some studies have explored the abnormalities in auditory brainstem responses (ABRs) when SSNHL occurs in MS [19,24,25,26,27,30]. Hearing loss in MS may be attributed to plaques occurring in the region where the cochlear nerve enters the brainstem, suggesting potential abnormalities in ABRs [30,34]. Typical findings include elongation in all waves except wave I, elongation in interpeak latencies of I–III and III–V waves, and changes in morphology or amplitude of the III and V waves [30,35]. In fact, such ABR abnormalities may not hold significant clinical meaning in patients already diagnosed with MS. In the case we presented, being a diagnosed MS case, we did not perform the ABR testing as its results were unlikely to impact the course of the disease. However, if SSNHL appears as the initial symptom of MS (as observed in all cases in Table 2), conducting an ABR test could prove valuable. Given the difficulty of suspecting MS as the primary cause of SSNHL, if the sudden loss fully recovers and other neurological symptoms persist, ABR testing may help differentiate the possibility of MS. Additionally, in idiopathic SSNHL, high-frequency loss often does not recover. However, among the cases in Table 2, three individuals exhibited distinct losses and successful recovery, specifically in the relatively higher frequency range above 2 kHz. While this pattern differs from idiopathic SSNHL, however, whether it could be considered a clinical indication of MS remains uncertain, requiring further accumulation of relevant data. Similar to ABRs, vestibular evoked myogenic potentials (VEMPs) can also be useful in the diagnosis of MS. A number of studies have shown the diagnostic value of VEMPs, particularly in detecting brainstem involvement in MS [36,37,38,39]. It is also worth implementing because it can be used to predict the prognosis of moderate to profound sensorineural hearing loss [40,41,42,43].

If a patient presents with concurrent ocular disease and hearing loss, Cogan syndrome is also a possibility. Cogan syndrome is characterized as a chronic inflammatory disorder commonly occurring in young adults, as in our case [44,45]. The occurrence of both ocular disease and inner ear disease aligns with the findings in our case [44,46,47]. However, our case differs in terms of the inner ear disease, as Cogan syndrome presents not only with hearing loss but also with dizziness resembling Meniere’s attacks and tends to exhibit more profound hearing loss [46,48]. Additionally, our case demonstrated relatively better speech discrimination, contrasting with the tendency for poor speech discrimination in Cogan syndrome cases [49]. Considering that anti-heat shock protein 70 (anti-Hsp70) can be identified in patients with sensorineural hearing loss, it may prove useful in the diagnosis of Cogan syndrome [50]. Based on these points, it becomes possible to differentiate between MS and Cogan syndrome.

## 4. Conclusions

SSNHL occurring in patients with MS tends to exhibit a more favorable treatment outcome compared with typical idiopathic SSNHL. The administration of systemic corticosteroids, a common approach for idiopathic SSNHL treatment, appears to be the most effective course of action in MS-associated SSNHL. Even in cases of bilateral involvement, the overall prognosis seems to align with the positive outcomes observed in unilateral SSNHL. This suggests that the management strategies employed for idiopathic SSNHL, particularly systemic corticosteroid therapy, can be beneficial for individuals experiencing SSNHL in MS.

## Figures and Tables

**Table 1:** Case series articles describing sudden hearing loss in multiple sclerosis.

Author (Year)	Sample Size	Age	Gender	Treatment Modality	Treatment Outcome	Recovery Period
Hellmann et al. (2011) [19]	Eleven	34.5 (17–52)	M: Four F: Seven	Three: No treatment Eight: IV methylprednisolone for 3–5 days, then a tapering course of oral prednisolone over 2 weeks	Ten: Recovered One: Not recovered	1–2 months
Leite et al. (2014) [29]	Seven	35.4 (21–51)	M: One F: Six	Three: No treatment Four: Pulse therapy of methylprednisolone	Among the three without treatment, two: partially recovered and one: not recovered; Among the four with treatment, three: fully recovered and one: not recovered	Not described

**Table 2:** Literature describing sudden hearing loss as an initial symptom of multiple sclerosis.

Author (Year)	Sample Size	Age	Gender	Side	Degree of the Loss	Treatment Modality	Treatment Outcome	Recovery Period
Shea and Brackman (1987) [19]	1	20	F	L	Profound loss at all frequencies	Prednisolone (60 mg/day) for 1 week and hydrochlorothiazide (50 mg/day)	Recovered	5 months
Drulović et al. (1994) [26]	2	33	F	L	50–60 dB	Not described	Recovered	1 months
		20	F	R	70–90 dB at 4–8 kHz	Corticosteroid	Recovered	14 days
Ozünlü et al. (1998) [21]	1	26	F	L	Profound loss (SDS 42%)	Corticosteroid (60 mg/day)	Recovered	16 days
Oh et al. (2008) [30] ^1^	1	46	F	L → R	Profound loss	Methylprednisolone (1 g/day) for 1 week	Lt: Recovered Rt: None	2 months
Anagnostouli et al. (2012) [24]	1	34	M	L	Not described	ITDI and IV methylprednisolone (1 g/day) for 5 days and tapering for 6 months	Recovered	8 months
Fernández-Menéndez et al. (2014) [31]	3	29	F	L	Not described	Five-day course of IV methylprednisolone (1 g/day)	Partially recovered	Not described
Valente et al. (2020) [32]	1	17	F	R	90 dB at 2–8 kHz	IV dexamethasone 8 mg and dihydrochloride 48 mg/day	Recovered	4 days
Cruz et al. (2022) [33]	4	26	M	R	65 dB at 3–8 kHz	Oral corticosteroid and then ITDI	Not recovered	Not described

^1^ Bilateral loss occurred sequentially from left to right ear.

**Table 3:** Literature addressing sudden hearing loss in patients diagnosed with multiple sclerosis.

Author (Year)	Sample Size	Age	Gender	Side	Degree of the Loss	Treatment Modality	Treatment Outcome	Recovery Period
Furman et al. (1989) [27]	1	25	F	R	Loss at 2–8 kHz SDS: 28%	Not described	Recovered	5 months
Yamasoba et al. (1997) [20]	1	30	M	L	22 dB SDS: 90%	Four courses of methylprednisolone (1 g/day for 3 days)	Recovered	4 months
Fernández-Menéndez et al. (2014) [31] ^1^	3	29	F	R	Not described	Five-day course of IV methylprednisolone (1 g/day)	Recovered	Not described
Tekin et al. (2014) [22]	1	30	F	R	113 dB	Prednisolone 1 mg/kg/day with tapering Hyperbaric oxygen therapy for 10 days	Recovered	3 months
Lee et al. (2019) [25]	1	28	M	R	30 dB at 2 kHz	Steroid pulse therapy	Recovered	1 month
Cruz et al. (2022) [33] ^2^	4	28	F	B	36.3 dB at Rt 40 dB at Lt	12-day steroid with a daily dose of 80 mg prednisolone	Recovered	4–6 weeks
		44	F	L	86.3 dB	Three-cycles of five-full-volume plasma exchange Six weekly doses of etanercept, anti-TNF agent	Partially recovered (51.3 dB)	6 weeks

^1^ In this document, a total of three cases are discussed, with one listed in Table 2 and one in Table 3. Another case could not be included in this review due to limited information provided in the literature. ^2^ This article discusses four cases, with one specified in Table 2 and two in Table 3. The remaining case could not be included due to limited information provided in the literature.

## Data Availability

Data sharing is not applicable to this article.
